# Supplementary material for: Predicting intentions towards long-term antidepressant use in the management of people with depression in primary care: A longitudinal survey study
Source: PLoS One. 2025 Mar 4;20(3):e0299676. doi: 10.1371/journal.pone.0299676 (PMC11878936; doi:10.1371/journal.pone.0299676)
Supplement: S1 File — (PDF) [file pone.0299676.s001.pdf]

Exploring patients' beliefs, attitudes, and behavioural intentions towards long-term depression management in primary care: A questionnaire survey study

Protocol

Rachel Ryves

University of Southampton

Funder: NIHR School for Primary Care Research

Sponsor: University of Southampton

ERGO ID: 25136

IRAS ID: 222680

Start Date: 01.06.2017

End Date: 30.04.2019

Depression and antidepressant treatment

Depression affects more than 1 in 10 adults in the UK [1], and mental ill-health represents between 9 and 23 percent of the health burden in the UK [2]. Southampton-led research using the CPRD database has shown that the prevalence of depression rose by only 3.9% between 2009 and 2013, while the number of antidepressant prescriptions rose by 36% over the same period [3], which is due to the rise of long-term use [4]. Some individuals with persistent long-term depression are defined within the NICE guidelines as having “subthreshold depression symptoms”, with insufficient other symptoms and/or functional impairment to meet the full diagnosis of major depression [5]. A systematic review found that continuing antidepressant treatment can reduce the risk of relapse in individuals with recurrent depression, however there is some uncertainty as to the length of treatment individuals with recurrent depression should stay on treatment for [6]. Therefore, while some long-term treatment of depression may be indicated, much of it may be inappropriate. A study of long-term antidepressant users concluded that a third to a half had no evidence-based indications to continue them, and could try stopping treatment [7]. However, many people taking antidepressants are prepared to continue indefinitely, due to fears of relapse and a perception that discontinuation would be a threat to their stability [8,9]. Moreover, a recent questionnaire survey study conducted in New Zealand found that individuals on long-term antidepressants had concerns about long-term adverse effects, including withdrawal effects, sexual problems, weight gain, feeling emotionally numb and the perception of being addicted to their medication [10]. Given the evidence that GPs are prescribing longer courses of antidepressant treatment, it has been recommended that guidelines need to include more information on how recurrent and long-term depression should be managed in primary care [11].

Long-term antidepressant use and review consultations

There is concern that few review consultations are carried out with patients who are long-term antidepressant users, with the percentage of patients reviewed during each year of antidepressant therapy decreasing over 10 years [12,13]. There are no formal processes within primary care for GPs to follow in order to carry out these reviews [13,14]. Reviewing long-term antidepressant use can reduce drug burden, with a primary care pharmacist-led study showing that around 15% of patients who had an active review had their antidepressant therapy altered, which in turn led to a reduction in antidepressant prescribing [14]. Qualitative research exploring primary care patients’ perceptions of the level of care they received for

their depression found that patients had a preference for collecting repeat prescriptions and were ambivalent about arranging follow-up consultations with their GP [15]. The authors suggest that GPs may need to play a bigger role in the patients' management of their depression and antidepressant use by encouraging patients to attend more face-to-face consultations to discuss treatment, long-term risks of antidepressant use, management, and support should they wish to discontinue treatment [10,15].

The importance of patients' illness beliefs

Given the burden of managing patients with depression in primary care, and with the rate of long-term antidepressant use on the rise [4], it is important to examine patients' beliefs about their illness, and their understanding of management strategies alternative to antidepressant use.

Lynch et al. explored whether illness beliefs predicted outcomes in depression [16,17], and findings showed that patients who had a stronger belief in the effectiveness of medication were more likely to be taking antidepressant treatment, more likely to believe that their condition had a chronic timeline, and more likely to be currently depressed [16]. Brown et al. (2001) also found that the illness perception of a 'chronic timeline' for depression was related to currently taking antidepressants [18]. Conversely, Lynch et al found that higher self-efficacy, and a belief that using talking therapies to manage depression, predicted improved PHQ-9 depression scores at follow-up. In addition, individuals who believed in engaging in activities such as exercise or keeping busy to treat their depression had improved depression outcomes at follow-up [10,16]. The prescription of antidepressants did not appear to mediate these relationships however [16]. Therefore patients' beliefs about treatments seem to be an important factor in determining the outcome of depression.

Links between beliefs, behaviours, and outcomes in depression

It is important to determine whether patients' beliefs predict specific behaviours, and whether these behaviours represent successful self-management.

The Theory of Planned Behaviour (TPB) is a psychosocial model of health behaviour (fig.1) that suggests attitudes towards behaviour, subjective norms, and perceived control of a specific behaviour can influence the intention of an individual to carry out this behaviour [19]. The TPB stipulates that more positive attitudes, higher normative expectations or expectations of significant others, and greater perceived behavioural control predict a greater intention and likelihood to carry out a behaviour. One possible way forward

therefore is to explore whether behavioural, normative, and control beliefs influence the intentions of patients to continue or stop their use of antidepressants for long-term depression, and whether these intentions are translated into actual behaviour.

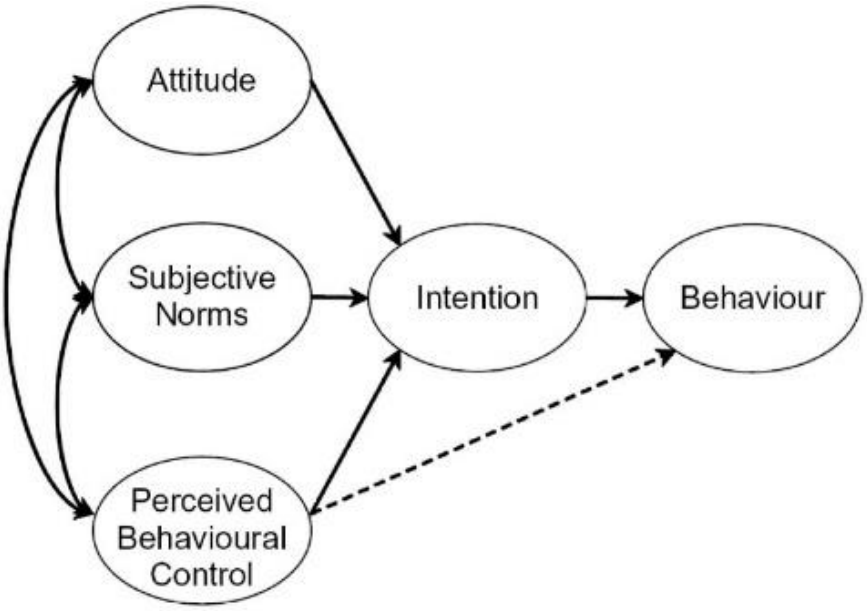

Figure 1. The Theory of Planned Behaviour

However, while the TPB may be useful in theoretically explaining health related behaviours, it has come under some criticism, including issues surrounding the intention-behaviour gap, whereby the TPB does not precisely explain how behavioural intentions predict actual behaviour. Moreover, the model has been criticised for its limited predictive validity and the methods used to test the theory [20,21]. One way in which psychologists have tackled these limitations to the model have been to use extended forms of the TPB by adding constructs in order to further explain how behavioural intentions may predict actual behaviour [20,21]. In terms of long-term depression management, further theories that may explain individuals' intentions to continue or discontinue antidepressant treatment or adopt specific management strategies.

Firstly, the Necessity-Concerns Framework (NCF) is a model that has been used to illustrate how patients' beliefs about a particular treatment influence the likelihood for adherence and engagement to this treatment [22]. A meta-analytic review of studies that used the Beliefs about Medicines Questionnaire (BMQ), developed from the NCF, found that higher adherence to taking medicines was associated with greater perceptions for the need for the medication, and smaller concerns about treatment [23]. This framework may therefore be useful in explaining why patients decide to stay on long-term antidepressant treatment, and

could add onto the constructs of the TPB. While the NCF is used to predict adherence to medications, it may be useful to explore what factors may influence discontinuation of prescribed medication.

A recent theory developed in polypharmacy research is Deprescribing Theory [24], which focuses on inappropriate medication use. A systematic review of studies focussing on medication withdrawal and patient beliefs about their medication use was conducted, and a theoretical model demonstrating barriers and enablers to deprescribing was created (figure 2.).

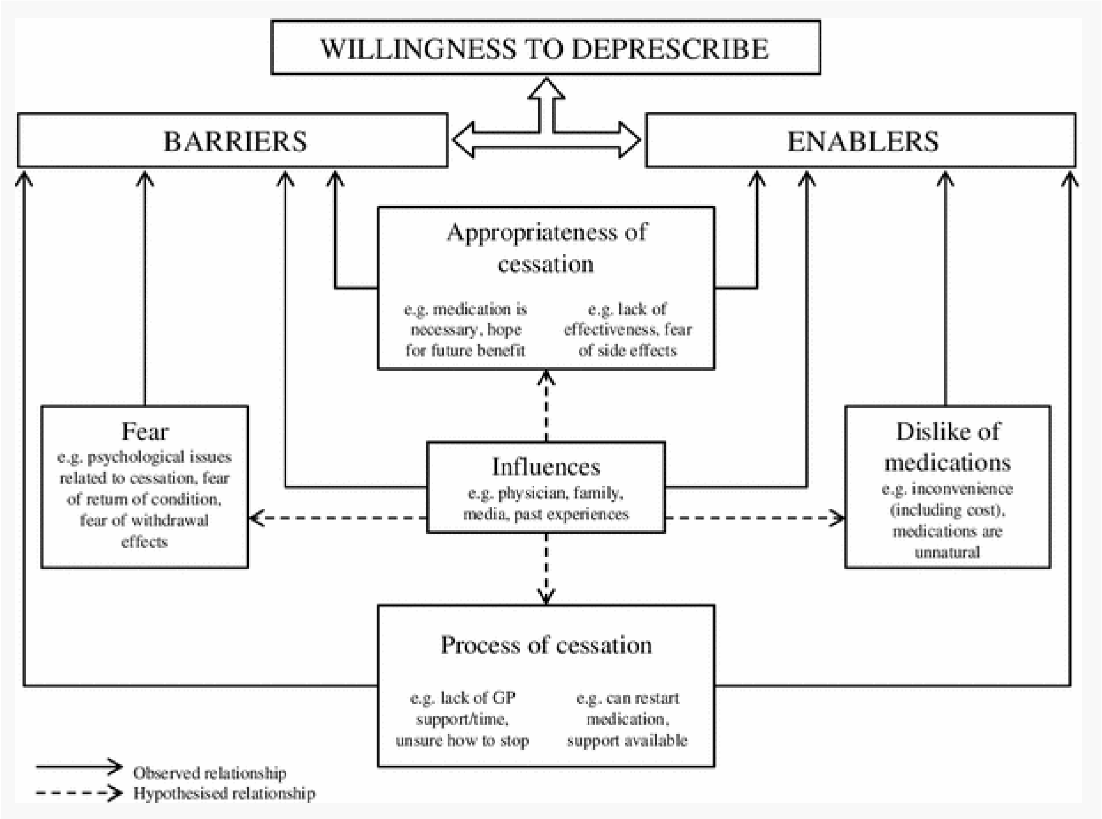

Figure 2. Deprescribing theory [24]

The theory suggests that a dislike of medication, appropriateness of stopping medication, and the influences and support of significant others may facilitate the discontinuation of unnecessary treatment. However, a lack of support, a belief that treatment is appropriate and past experiences may in turn act as a barrier to stopping treatment, in conjunction with the fear related to treatment cessation. As with the NCF, this theory may give additional insight into the psychosocial predictors of intentions to continue or discontinue antidepressant use.

Aims

The overall aim of the PhD is to explore patients' beliefs, attitudes, and behavioural intentions towards long-term depression management and long-term antidepressant use in primary care.

The study has two aims:

- to investigate whether particular psychosocial factors predict the intentions of individuals with long-term depression to continue or stop their use of antidepressants, and whether these intentions are translated into actual behaviour.
- to elicit patient beliefs about long-term antidepressant use and long-term depression management in primary care.

Design

Embedded mixed methods study. A quantitative cross-sectional questionnaire survey study will conducted, with a sequential nested qualitative study.

Hypotheses

Patients with greater behavioural, normative, and control beliefs about stopping antidepressants for their long-term depression will have greater intentions to discontinue treatment, and be more likely to carry out this behaviour.

Patients with greater beliefs in the need for antidepressants for their long-term depression, and fewer concerns about the consequences of taking them, will have less intention towards discontinuing their medication, and will be less likely to stop antidepressant treatment.

Setting

Primary Care practices in Hampshire, Dorset, Wiltshire, and the West of England, accessed via CRN: Wessex.

Sample

Practices

Research active primary care practices in the local NIHR funded clinical research network (CRN Wessex) will be recruited to the study. The study will be eligible for registration on the NIHR CRN portfolio, as it is funded through an NIHR SPCR studentship.

Participants

Participants will be 400 patients from primary care practices. This sample size should be suitable for data analysis (structural equation modelling). While there is no set calculation for determining a sample size where structural equation modelling is used, the literature suggests that a sample of between 200-400 participants is sufficient [25-27]. It is also anticipated that this sample size will be feasible in a primary care setting.

Patients will be eligible to take part if they:

- are 18 years old and over;
- have been receiving antidepressant prescriptions for 2 years or longer. (According to the DSM-5, long-term depression is classified as experiencing symptoms of depression for at least 2 years [28]).

Patients will not be excluded based on their level of depression. Patients with comorbid physical conditions will not be excluded.

Patients will be excluded if they:

- are primarily using antidepressants for treatment of conditions other than depression (e.g. tricyclic antidepressant use for pain);
- mainly have their depression managed in secondary care;
- have a serious psychiatric condition (e.g. psychosis, comorbid dementia, significant substance misuse, PTSD) that makes depression a secondary rather than primary diagnosis.
- are at risk of suicide/self-harm and need urgent referral to secondary care.
- are terminally ill, lacking capacity.
- are deemed unsuitable after screening by a GP.

Identifying participants

Practices will be required to conduct a database search to identify patients according to the eligibility criteria listed above. Practices will be provided with a list of Read Codes to conduct the search in order to minimise the risk of missing eligible patients and to ensure consistent searching strategies between practices. GPs will screen the list of eligible patients to screen any that are deemed unsuitable to contact as per the exclusion criteria above.

Recruitment

Patients identified through the database search will be sent a pack by the practice. Each pack will have a unique ID code attributed to it. Practices will be required to make a note of the ID number on the records of patients that have been sent a questionnaire booklet.

The pack will include:

- A cover letter (on practice headed paper) inviting patients to take part in the study.
- A patient information leaflet (PIL) providing details about the study.
- A questionnaire booklet.
- A consent form.
- 2 FREEPOST envelopes.

If patients are interested in taking part in the study, they will have the option to complete the questionnaires either by post, or online.

Consent

As per HRA guidance, completion and returning of the postal/online questionnaire to the researcher indicates consent on behalf of the participant. The PIL will include a lay summary highlighting the purpose of the research, and why patients have been approached. It will also include details on what the participant is required to do, and information about the ethics and governance of the study.

Participants will have the option to contact the researcher by telephone to have any additional queries they may have about the study to be answered before completing the questionnaires.

Participants will be required to complete a consent form to say they are happy for their GP practice to complete a notes review at six months, to see whether they have been for an appointment to review their antidepressant use and/or begun discontinuation of their antidepressant medication. Participants will also need to give consent should they wish to be contacted about taking part in the qualitative interview study. This consent form will be returned separately to the questionnaire booklet, for data protection purposes.

Postal Questionnaires

Participants that complete the questionnaire booklet will be required to complete it and return it using one of the FREEPOST envelopes provided. Each questionnaire booklet will have a unique participant ID number. Once the researcher has received the completed

questionnaire booklet, they will notify the practice that the patient has completed the questionnaire, and to mark this on their record, so that a notes review can be conducted at 6 months.

Online Questionnaires

Alternatively, online questionnaires will be available for participants via the University of Southampton's iSurvey website (www.isurvey.soton.ac.uk). Participants who would like to complete the questionnaires online will be able to log onto the website and register using a unique participant number, which will be provided on the cover letter. Once logged on, participants will have another opportunity to read through the information leaflet before continuing to complete the questionnaires. As with the postal questionnaires that are returned, the researcher will notify the relevant practice once a patient has completed the questionnaire online, so that this can be marked on their patient record.

Follow-up letters

If participants do not return questionnaires within 2 months of receiving the packs, a letter will be sent out via the practices, to remind them about the study. The follow-up letter will remind them of the purpose of the study, and invite them to request another copy of the questionnaire (should they require one), return the paper questionnaire, or complete it online. The letter will include their participant ID number for reference.

Research Questionnaires

Beliefs about long-term antidepressant use questionnaire

The bespoke questionnaire has been created based on the constructs of the TPB and other theoretical frameworks (deprescribing theory [24] and the necessity/concerns framework [23]). A manual has been used to guide the development of questionnaire items to measure attitudes, normative beliefs, control beliefs, and intentions towards long-term antidepressant use [29]. The Beliefs about Medicines Questionnaire –Specific (BMQ), a validated questionnaire asking patients about their beliefs about the medicines they are taking [30] is included in the questionnaire, with items tailored to be specific to antidepressant use. Moreover, some items from the Patient Attitudes Towards Deprescribing (PATD) Questionnaire, a validated measure of patients' beliefs about deprescribing [28] have been used to ask participants about their views on stopping antidepressant treatment.

The questionnaire survey has been developed and refined through testing in cognitive interviews, involving participants with long-term depression and in receipt of antidepressant treatment.

Research Questionnaires

Participants will also be required to complete some additional questionnaires:

- The Beliefs about Depression questionnaire [16], a 48-item questionnaire that measures illness beliefs about depression;
- The PHQ-8 questionnaire [31], an 8-item questionnaire to measure current symptoms and severity of depression;
- A bespoke past history of depression questionnaire, to measure the duration of the participant's depression and duration of antidepressant treatment;
- A bespoke demographic questionnaire.

Notes review

Participants will be asked if they consent for their medical records to be accessed by the researcher 6 months after their involvement in the study, in order to conduct a notes review. The notes review will be carried out in order to measure the proportion of participants who attended a review consultation for their depression, and are in the process of discontinuing antidepressant treatment, which will be indicated by a change in their antidepressant dosage.

Analysis

Structural equation modelling will be conducted on the data collected from the questionnaires. Path analysis will test the ability of the TPB along with additional constructs from the NCF and Deprescribing Theory to predict patients' intentions to stop or continue antidepressant treatment for their long-term depression. Moreover, it will be possible to determine whether certain constructs within the model are better at determining patient behaviour towards long-term antidepressant use. Bootstrapping will be used if there are issues with recruitment resulting in a small sample size.

Nested Qualitative Study

Aim

A nested qualitative study will be carried out in order to explore patients' views and understanding of the management of long-term depression and long-term antidepressant use for depression in primary care.

Design

Semi-structured, face-to-face or telephone interviews.

Sample

Participants who have completed the questionnaires will have the option to consent to being contacted about taking part in a one-off interview with the researcher.

Up to 40 patients will be interviewed for the qualitative study. Participants will be purposively sampled based on their demographic characteristics, as well as their responses to the questionnaires. Using maximum variation sampling will enable heterogeneity, in order to understand the views and understanding of long-term depression management in primary care from a more diverse sample of people. Recruitment will continue until data has reached saturation.

Procedure

The researcher will make contact with patients and arrange for a time that is convenient to them to be interviewed. Patients will have the option to be interviewed either at their GP surgery or their own home, or via telephone. Patients will have the opportunity to read through an information leaflet providing details of the study, and will be asked to provide written, informed consent prior to taking part.

The qualitative interviews will be semi-structured and based around a topic guide consisting of open-ended questions that will prompt participants about their views and experiences of long-term depression, its management in primary care, and long-term antidepressant use. The use of a semi-structured, open-ended topic guide will allow for consistency of questions asked between interviews, but will provide an opportunity for further exploration of new ideas arising from interviews.

Interviews will be recorded and transcribed verbatim and imported into NVivo 11 software for analysis.

Analysis

Inductive thematic analysis [32] of the data will be carried out in order to derive sub-themes and themes related to the study questions. Inductive thematic analysis is appropriate as its methods will allow for patterns and associations to be identified within and between the data, in order to give some understanding towards long-term depression management in primary care. The analysis will follow the 6 steps advised by Braun & Clarke (2006):

1) Familiarisation of the data, by repeated reading of interview transcripts, and searching for meaning and patterns.
2) Generation of initial codes, to highlight findings of interest within the data.
3) Development of potential themes, and allocation of codes to these themes.
4) Reviewing and refining the themes, in terms of well they explain the findings within the data set.
5) Defining and naming the themes, to explain what each theme is about and to form a narrative of the analysis.
6) The reporting of the findings.

An iterative approach will be adopted during the data collection and preliminary analysis of the findings, using a constant comparison approach. Any newly collected data collected will be compared with data from previous interviews, to create themes that relate to the research question.

In order to facilitate the analytic process, a coding manual will be used to define and provide illustrative examples of the codes. Meetings will be held with members of the supervisory team (who have expertise in qualitative research) in order to discuss analytic findings and to identify and resolve any discrepancies between the data and themes created. Field notes will be written after each interview to keep track of initial thoughts and points of interest from the interviews, and a reflexive journal will also be kept in order to note the influence of the researcher's beliefs and behaviours on the research process.

References

1. Sweet D. Health. *Social Trends* 2011;41:1-36.
2. Department of Health. *Healthy lives, healthy people: Our strategy for public health in England*; 2010.
3. Kendrick T, Stuart B, Newell C, et al. Changes in rates of recorded depression in English primary care 2003–2013: Time trend analyses of effects of the economic recession, and the GP contract quality outcomes framework (QOF). *Journal of Affective Disorders* 2015;180:68-78.
4. Moore M, Yuen HM, Dunn N, et al. Explaining the rise in antidepressant prescribing: a descriptive study using the general practice research database. *BMJ: British Medical Journal* 2009;339(b3999):1-7.
5. NICE. Depression: The treatment and management of depression in adults (updated edition). *National Institute for Health and Clinical Excellence: Guidance* 2010.
6. Geddes JR, Carney SM, Davies C, et al. Relapse prevention with antidepressant drug treatment in depressive disorders: a systematic review. *The Lancet* 2003;361(9358):653-61.
7. Cruickshank G, MacGillivray S, Bruce D, et al. Cross-sectional survey of patients in receipt of long-term repeat prescriptions for antidepressant drugs in primary care. *Mental health in family medicine* 2008;5(2).
8. Leydon GM, Rodgers L, Kendrick T. A qualitative study of patient views on discontinuing long-term selective serotonin reuptake inhibitors. *Family practice* 2007;24(6):570-75.
9. Dickinson R, Knapp P, House AO, et al. Long-term prescribing of antidepressants in the older population: a qualitative study. *Br J Gen Pract* 2010;60(573):e144-e55.
10. Cartwright C, Gibson K, Read J, et al. Long-term antidepressant use: patient perspectives of benefits and adverse effects. *Patient preference and adherence* 2016;10:1401-07.
11. Kendrick T, Stuart B, Newell C, et al. Did NICE guidelines and the Quality Outcomes Framework change GP antidepressant prescribing in England? Observational study with time trend analyses 2003–2013. *Journal of Affective Disorders* 2015;186:171-77.
12. Middleton DJ, Cameron IM, Reid IC. Continuity and monitoring of antidepressant therapy in a primary care setting. *Quality in Primary Care* 2011;19(2):109-13.
13. Sinclair JE, Aucott LS, Lawton K, et al. The monitoring of longer term prescriptions of antidepressants: observational study in a primary care setting. *Fam Pract* 2014;31(4):419-26.
14. Johnson CF, Macdonald HJ, Atkinson P, et al. Reviewing long-term antidepressants can reduce drug burden: a prospective observational cohort study. *Br J Gen Pract* 2012;62(604):e773-9.
15. Gask L, Rogers A, Oliver D, et al. Qualitative study of patients' perceptions of the quality of care for depression in general practice. *British Journal of General Practice* 2003;53(489):278-83.

16. Lynch J, Moore M, Moss-Morris R, et al. Do patients' illness beliefs predict depression measures at six months in primary care; a longitudinal study. *Journal of Affective Disorders* 2015;174:665-71.
17. Lynch J, Moore M, Moss-Morris R, et al. Are patient beliefs important in determining adherence to treatment and outcome for depression? Development of the beliefs about depression questionnaire. *Journal of affective disorders* 2011;133(1–2):29-41.
18. Brown C, Dunbar-Jacob J, Palenchar DR, et al. Primary care patients' personal illness models for depression: a preliminary investigation. *Family practice* 2001;18(3):314-20.
19. Ajzen I. The Theory of Planned Behaviour. *Organizational Behaviour and Human Decision Processes* 1991;50:179-211.
20. Sniehotta FF, Presseau J, Araújo-Soares V. Time to retire the theory of planned behaviour. *Health Psychology Review* 2014;8(1):1-7.
21. Ogden J. *Health Psychology: A textbook*. Maidenhead: Open University Press; 2012.
22. Horne R, Weinman J. Patients' beliefs about prescribed medicines and their role in adherence to treatment in chronic physical illness. *J Psychosom Res* 1999;47(6):555-67.
23. Horne R, Chapman SC, Parham R, et al. Understanding patients' adherence-related beliefs about medicines prescribed for long-term conditions: a meta-analytic review of the Necessity-Concerns Framework. *PloS one* 2013;8(12):e80633.
24. Reeve E, To J, Hendrix I, et al. Patient barriers to and enablers of deprescribing: a systematic review. *Drugs and Aging* 2013;30(10):793-807.
25. Breckler SJ. Applications of covariance structure modeling in psychology: Cause for concern? *Psychological bulletin* 1990;107(2):260.
26. Kline RB. *Principles and practice of structural equation modeling*: Guilford publications; 2015.
27. Shah R, Goldstein SM. Use of structural equation modeling in operations management research: Looking back and forward. *Journal of Operations Management* 2006;24(2):148-69.
28. Pfoh ER, Mojtabai R, Bailey J, et al. Conformance to Depression Process Measures of Medicare Part B Beneficiaries in Primary Care Settings. *Journal of the American Geriatrics Society* 2015;63(7):1338-45 8p.
29. Francis JJ, Eccles MP, Johnston M, et al. Constructing questionnaires based on the theory of planned behaviour. *A manual for health services researchers* 2004;2010:2-12.
30. Horne R, Weinman J, Hankins M. The beliefs about medicines questionnaire: The development and evaluation of a new method for assessing the cognitive representation of medication. *Psychology & Health* 1999;14(1):1-24.
31. Kroenke K, Spitzer RL, Williams JB. The Phq ‐ 9. *Journal of general internal medicine* 2001;16(9):606-13.
32. Braun V, Clarke V. Using thematic analysis in psychology. *Qualitative research in psychology* 2006;3(2):77-101.
