# Supplementary material for: Predicting intentions towards long-term antidepressant use in the management of people with depression in primary care: A longitudinal survey study
Source: PLoS One. 2025 Mar 4;20(3):e0299676. doi: 10.1371/journal.pone.0299676 (PMC11878936; doi:10.1371/journal.pone.0299676)
Supplement: S2 File — (PDF) [file pone.0299676.s002.pdf]

UNIVERSITY OF Southampton

APPLAUD

Attitudes and Preferences of People regarding Long-term Antidepressant Use for Depression

Questionnaire Survey

Patient ID Number

Please ensure you have read the information leaflet included in the pack before you decide to take part.

The aim of this study is to look at what people think and do about their long-term depression, anxiety, or low mood.

You will be asked questions about your beliefs about your depression, anxiety, and low mood; your antidepressant use; and current symptoms of depression, anxiety, or low mood. Completing the questionnaires should take no longer than 30 minutes.

Your GP practice will be notified that you have taken part in the study. **The answers you give in the questionnaires will not be shared with your GP and will only be used for the purpose of this study.**

When you have completed the questionnaire, please return it in the FREEPOST Envelope provided, or to:

The APPLAUD Study
FREEPOST LICENCE NO: RTLE-TKTU-KRBX
Primary Care & Population Sciences
Aldermoor Health Centre
Aldermoor Close
Southampton
SO16 5ST

Online Questionnaire login details:

If you would prefer to complete the questionnaire online, please go to www.isurvey.soton.ac.uk/24432

Once you have gone to the website, please log in using your patient ID number in the box above, and using the password: **APPLAUD1718**

Socio-demographic Questionnaire

Please answer ALL questions as fully as you can, ticking the box next to the answer that applies to you most.

1. Gender

Male ☐

Female ☐

2. Date of Birth

D	D		M	M		Y	Y

3. Ethnic Group

White ☐	White Other ☐	Black Caribbean ☐	Black African ☐
Black other ☐	Indian ☐	Pakistani ☐	
Bangladeshi ☐	Chinese ☐	Other Asian group ☐	

If "White other", "Other Asian group", or "Black other" please specify: ______

4. Marital Status

Married ☐ | Cohabiting ☐ | Widowed ☐ | Separated ☐ | Divorced ☐ | Single ☐

5. Dependants

Number of dependants (over 17) ☐

Number of children under 5 ☐

Number of children 5-16 inclusive ☐

6. Accommodation status

Owner-occupied ☐	Private rental ☐
Job related ☐	Lives with parents ☐
Council/housing association ☐	Other ☐

If "Other" please specify: ______

7. Type of accommodation

Detached ☐	Semi-detached ☐	End-terrace ☐	Other ☐
Mid-terrace ☐	Flat/Maisonette ☐	Bedsit ☐	
Hostel ☐	Halls of residence ☐	No fixed abode ☐	

If "Other" please specify: ______

8. Still in education

No ☐ Yes FT ☐ Yes PT ☐

If No, please answer question 8a.

If Yes, please answer question 8b.

8a. Age left full-time education

8b. Course title

9. Highest exam level

- None ☐
- CSE/NVQ Level 1 ☐
- GCSE/O Level/NVQ Level 2 ☐
- A level/BTEC/NVQ Level 3 ☐
- HNC/HND/City & Guilds/Teaching qualification/NVQ Level 4 ☐
- Degree/higher degree/NVQ Level 5 ☐
- Vocational qualification ☐
- Other ☐

If "Other" or unsure of level, enter here:

10. Economic position

Full-time work ☐	Part-time work ☐	Self-employed ☐	Other ☐
Voluntary work ☐	Unemployed ☐	Permanently sick/disabled ☐	
Homemaker ☐	Retired ☐	Student ☐	

If "Other", please specify:

11. Occupation

Please state your occupation:

Function of organisation/nature of business:

APPLAUD Questionnaire

This questionnaire asks you about your antidepressant use. Please answer the questions as honestly as you can. Your doctor will <u>not</u> see the results of the questionnaire.

Please read each statement carefully, and circle the number of the response that you feel applies most to you.

1. I *<u>expect</u>* to start to come off antidepressants within the next six months						
**Strongly Disagree**						**Strongly Agree**
1	2	3	4	5	6	7

2. I *<u>want</u>* to start to come off antidepressants within the next six months						
**Strongly Disagree**						**Strongly Agree**
1	2	3	4	5	6	7

3. I *<u>intend</u>* to start to come off antidepressants within the next six months						
**Strongly Disagree**						**Strongly Agree**
1	2	3	4	5	6	7

4. I am confident that I could start to come off antidepressants within the next six months, if I wanted to						
**Strongly Disagree**						**Strongly Agree**
1	2	3	4	5	6	7

5. The decision for me to start to come off antidepressants is beyond my control						
**Strongly Disagree**						**Strongly Agree**
1	2	3	4	5	6	7

6. For *<u>me</u>*, to start to come off antidepressants within the next six months would be:								
**Desirable**	1	2	3	4	5	6	7	**Undesirable**
**Unnecessary**	1	2	3	4	5	6	7	**Necessary**
**Beneficial**	1	2	3	4	5	6	7	**Harmful**
**Good**	1	2	3	4	5	6	7	**Bad**
**Unpleasant**	1	2	3	4	5	6	7	**Pleasant**
**Safe**	1	2	3	4	5	6	7	**Dangerous**
**Easy**	1	2	3	4	5	6	7	**Difficult**

7. Whether I start to come off antidepressants within the next six months or not is entirely up to me						
**Strongly Disagree**						**Strongly Agree**
1	2	3	4	5	6	7

8. Most people who are important to me think that I:								
**should**	1	2	3	4	5	6	7	**should not**
start to come off antidepressants								start to come off antidepressants

9. I feel under social pressure to start to come off antidepressants within the next six months						
**Strongly Disagree**						**Strongly Agree**
1	2	3	4	5	6	7

10. My doctor(s) think that I should start to come off antidepressants within the next six months						
**Strongly Disagree**						**Strongly Agree**
1	2	3	4	5	6	7

11. People who are close to me want me to start to come off antidepressants within the next six months						
**Strongly Disagree**						**Strongly Agree**
1	2	3	4	5	6	7

Please read each of the following statements carefully, and circle your response to how much you agree or disagree with the statements.

12. My health, at present, depends on my antidepressants				
Strongly agree	Agree	Uncertain	Disagree	Strongly disagree

13. Having to take antidepressants worries me				
Strongly agree	Agree	Uncertain	Disagree	Strongly disagree

14. My life would be impossible without my antidepressants				
Strongly agree	Agree	Uncertain	Disagree	Strongly disagree

15.Without my antidepressants I would be very ill				
Strongly agree	Agree	Uncertain	Disagree	Strongly disagree

16. I sometimes worry about long-term effects of my antidepressants				
Strongly agree	Agree	Uncertain	Disagree	Strongly disagree

17. My antidepressants are a mystery to me				
Strongly agree	Agree	Uncertain	Disagree	Strongly disagree

18. My health in the future will depend on my antidepressants				
Strongly agree	Agree	Uncertain	Disagree	Strongly disagree

19. My antidepressants disrupt my life				
Strongly agree	Agree	Uncertain	Disagree	Strongly disagree

20. I sometimes worry about becoming too dependent on my antidepressants				
Strongly agree	Agree	Uncertain	Disagree	Strongly disagree

21. My antidepressants protect me from becoming worse				
Strongly agree	Agree	Uncertain	Disagree	Strongly disagree

Please read through the following statements carefully, and indicate whether you agree with them by ticking the appropriate box.

	Strongly agree	Agree	Unsure	Disagree	Strongly disagree
22. I am comfortable taking antidepressants					
23. I believe that my antidepressants are necessary					
24. If my doctor said it was possible I would be willing to stop taking my antidepressants					
25. I would like to stop taking my antidepressants					
26. I feel I may be taking antidepressants that I no longer need					
27. I would accept managing my depression in other ways					
28. I have a good understanding of the reasons I was prescribed antidepressants					
29. Not having to pay for prescriptions would play a role in my willingness to stop taking antidepressants					
30. I believe my antidepressants are giving me side effects					

31. Have you ever tried to stop taking antidepressants *with* your doctor's knowledge?

Yes ☐ No ☐

32. Have you ever tried to stop taking antidepressants *without* your doctor's knowledge?

Yes ☐ No ☐

33. How comfortable would you be if the following health professionals were involved in stopping your antidepressants and provided the follow up? *(Please tick your answer)*			
	Uncomfortable	Unsure	Comfortable
Doctor			
Nurse Practitioner			
Pharmacist			

34. If your antidepressants were stopped, what follow-up would you like? *(Please tick all that apply)*	
	Face-to-face appointment with my doctor
	Face-to-face appointment with a practice nurse
	Face-to-face appointments with a pharmacist
	Phone call(s) from my doctor
	Phone call(s) from a practice nurse
	Phone call(s) from a pharmacist
	Written information via post
	Written information via email
	I wouldn’t need follow-up. I would be happy contacting a health professional if I had any problems

The next question is optional. If you have any additional comments that you feel may be useful for the researcher to know, then please write them in the box below:

35. Additional comments:

Beliefs about Depression Questionnaire

We are interested in finding out about <u>your</u> beliefs about your condition. We are interested in <u>your</u> beliefs rather than those of your health professional, family member or friends. For each of the following questions please put a tick in the box which best represents your beliefs.

1. Your Doctor has diagnosed you with depression. Do you think this is the correct name for your condition?

Yes		No	

If no, what would **<u>you</u>** call your condition? ______________________________

2. Which of the following symptoms do you think are related to your depression?

Symptom	Yes	No	Symptom	Yes	No
Lack of hope for the future			Reduced energy		
Pain			Tiredness		
Feeling of a black cloud hanging over me			Dizziness		
Changes in appetite			Weight loss		
Breathlessness			Unable to enjoy things		
Agitation			Muscle aches		
Suicidal thoughts			Short tempered		
Other symptoms, please specify: ………………………………………………..					

For each of the following statements please indicate how much you agree or disagree by placing a tick in the relevant box. There are no correct answers – we want to know what **<u>you</u>** think.

3. What do you think **caused** your depression/condition?

	Strongly disagree	Moderately disagree	Slightly disagree	Slightly agree	Moderately agree	Strongly agree
Low esteem/ lack of confidence						
My personal flaws						
Unresolved problems from the past						
Problems from childhood						
Problems with relationships (family, partner or friends)						
Bereavement						
Work						
Physical illness						
Chemical or hormonal changes						
Inherited/ caused by genetic factors						
Overdoing things						
I don't know what caused my depression						
Other causes of my condition, please specify: ……………………………………………………………….						

4. How **long** do you think this condition will **last**?

	Strongly disagree	Moderately disagree	Slightly disagree	Slightly agree	Moderately agree	Strongly agree
I will always have this condition						
My symptoms come and go in cycles						
I expect to have this condition for the rest of my life						
I go through cycles in which my condition gets better and worse						

5. What do you think would help **control** or **cure** your condition?

	Strongly disagree	Moderately disagree	Slightly disagree	Slightly agree	Moderately agree	Strongly agree
Medication prescribed by my doctor						
Changing how I think about myself						
Changing my behaviour						
Spirituality/ religious beliefs						
I cannot do anything to alter the course of my condition						
I don't know what will help						
Counselling/ therapy						
Talking/ support from family or friends						
Talking/ support from professionals						
Talking/ support from fellow sufferers						
Keeping busy						
Medication prescribed by another practitioner e.g. homeopath						
Natural medicines e.g. St John's Wort						
Exercise						
Other things which help my condition, please specify: …………………………………………………………………………						

6. What are the **consequences** of having this condition?

	Strongly disagree	Moderately disagree	Slightly disagree	Slightly agree	Moderately agree	Strongly agree
I do not want to go out						
I neglect myself						
I have to hide how I feel from other people						
I want to avoid other people						
Having this condition makes me a stronger person						
There is a stigma						
My condition affects how others see me						
Other consequences of my condition, please specify: …………………………………………………………………………						

PHQ – 8 Questionnaire

**Over the <u>last 2 weeks</u>, how often have you been bothered by any of the following problems?**	**Not at all**	**Several days**	**More than half the days**	**Nearly every day**
Little interest or pleasure in doing things	☐	☐	☐	☐
Feeling down, depressed, or hopeless	☐	☐	☐	☐
Trouble falling or staying asleep, or sleeping too much	☐	☐	☐	☐
Feeling tired or having little energy	☐	☐	☐	☐
Poor appetite or overeating	☐	☐	☐	☐
Feeling bad about yourself – or that you are a failure or have let yourself or your family down	☐	☐	☐	☐
Trouble concentrating on things, such as reading the newspaper or watching television	☐	☐	☐	☐
Moving or speaking so slowly that other people could have noticed? Or the opposite – being so fidgety or restless that you have been moving around a lot more than usual	☐	☐	☐	☐

Past History of Depression Questionnaire

Please answer <u>ALL</u> questions as fully as you can, putting your answers in the boxes provided.

1. **How long have you suffered from depression?**

   ☐ Years ☐ Months

2. **How long have you been taking antidepressants for your current episode of depression?**

   ☐ Years ☐ Months

3. **What antidepressant are you currently taking?** *(Name of drug and dose if known)*

   ☐

4. **How old were you when were you first prescribed antidepressants?**

   ☐ Years

5. **Have you successfully stopped antidepressant treatment before?**
   (Successfully = experienced symptom free episode(s) while off antidepressant treatment)

   ☐ Yes
   ☐ No

   If yes, how long were you off antidepressants for?

   ☐ Years ☐ Months

Thank you for taking part in this questionnaire study

Study Aims

The aim of this study is to investigate whether particular factors influence the intentions of individuals with long-term depression, anxiety or low mood to continue or stop their use of antidepressants, and whether these intentions become an actual behaviour.

You were asked to complete a set of questionnaires that asked you about your beliefs about long-term antidepressant use, your current symptoms of depression, anxiety, low mood and quality of life, and your history of antidepressant use. The information you provided will be used to see whether certain attitudes, behaviours, and beliefs about long-term antidepressant use for long-term depression, anxiety or low mood predict whether people stop or continue taking antidepressants.

Future contact

If you would like to receive a summary of results from the main study, then please contact the researcher. It is anticipated that the PhD will be completed by 2019. You may also request to be notified of any publications relating to the study.

If you have any questions about the study, then please feel free to contact Rachel Ryves via email (applaud@soton.ac.uk) or telephone (02380 591 755 / 07379523467). If you have any concerns about the study, then please contact Rachel’s Lead PhD Supervisor, Professor Tony Kendrick (a.r.kendrick@soton.ac.uk; 02380 591 790). You may also contact the Research Integrity & Governance Team at the University of Southampton (email rgoinfo@soton.ac.uk or telephone 02380595058).

Seeking Medical Advice

If you feel that your involvement in the study has caused concern or raised questions about your depression or your treatment, then we strongly recommend that you see your doctor.

Sources of additional information and support:

http://www.nhs.uk/livewell/mentalhealth/Pages/Mentalhealthhome.aspx

www.mind.org.uk/information-support/

Medicine

UNIVERSITY OF Southampton

CONSENT FORM

Title of Project: Attitudes and Preferences of People regarding Long-term Antidepressant Use (The APPLAUD Study)

Name of Researcher: Rachel Ryves

IRAS ID: 222680

Study Number: 25136

Participant Identification Number for this trial:

The consent form asks if you consent for your doctor to look your medical records. This is to see if you have been to see a GP for your depression, anxiety or low mood within 6 months of completing the questionnaires; and whether you have continued or stopped your antidepressants by that point. The GP would then let the researcher know this information by completing a form and sending it to the researcher. No other medical or personal information will be shared with the researcher.

Please **initial** box

1. I understand that relevant sections of my medical notes and data collected during the study may be looked at by individuals from University of Southampton, from regulatory authorities or from the NHS Trust, where it is relevant to my taking part in this research. I give permission for these individuals to have access to my records. ☐

2. I agree to my General Practitioner being involved in the study, including any necessary exchange of information about me between my GP and the research team. ☐

3. I agree to be contacted about taking part in the interview study. **I understand that this part of the is optional**, and that not all participants who express an interest will be interviewed. ☐

____________________	____________________	____________________
Name of Participant	Date	Signature

PLEASE TURN OVER

Medicine

UNIVERSITY OF Southampton

If you are happy about being contacted about taking part in the interview study, please fill in the form below. This part of the study is optional.

The APPLAUD Study: CONSENT TO CONTACT FORM Study ID: 25136

Participant ID:

Name: ____

Telephone Number: ____

Email Address: ____

Preferred method of contact: Email Phone
(please circle all that apply):

Preferred day to be contacted: Mon Tues Weds Thurs Fri Sat Sun
(please circle all that apply):

Preferred contact time: 9 am -12pm 12pm – 2pm 2pm – 5pm 5pm – 8pm
(please circle all that apply)

PLEASE RETURN THIS FORM IN THE SMALL FREEPOST ENVELOPE

Thank you.
