# Supplementary material for: Predicting intentions towards long-term antidepressant use in the management of people with depression in primary care: A longitudinal survey study
Source: PLoS One. 2025 Mar 4;20(3):e0299676. doi: 10.1371/journal.pone.0299676 (PMC11878936; doi:10.1371/journal.pone.0299676)
Supplement: S3 File — (PDF) [file pone.0299676.s003.pdf]

UNIVERSITY OF Southampton

Attitudes and Preferences of People regarding Long-term Antidepressant Use for Depression
Notes Review

Name of Researcher: Rachel Dewar-Haggart

Participant ID Number:

Please record all prescribed antidepressant medications during the 6-month timeframe in the boxes below (if dose has changed please list as a separate medication):

N.B – Please check the 6-month timeframe for this patient on the Participant Information for Notes Review document.

	Name of prescription	Dosage (mgs.)	Date Prescribed	If repeat, how requested?
**1**				During appointment ☐<br>Through reception ☐<br>Online ☐
**2**				During appointment ☐<br>Through reception ☐<br>Online ☐
**3**				During appointment ☐<br>Through reception ☐<br>Online ☐
**4**				During appointment ☐<br>Through reception ☐<br>Online ☐
**5**				During appointment ☐<br>Through reception ☐<br>Online ☐
**6**				During appointment ☐<br>Through reception ☐<br>Online ☐
**7**				During appointment ☐<br>Through reception ☐<br>Online ☐

**8**				During appointment<br>Through reception<br>Online	☐<br>☐<br>☐
**9**				During appointment<br>Through reception<br>Online	☐<br>☐<br>☐
**10**				During appointment<br>Through reception<br>Online	☐<br>☐<br>☐

Please give details of any of the following services that have been accessed by the participant in the last 6 months.

PLEASE ONLY INCLUDE APPOINTMENTS THAT CONCERNED THE PATIENT'S **MENTAL HEALTH CARE**.

**Service**	**Accessed?**	**No. Contacts in last 6 months**	**Date(s) of access**
**General Practitioner (face to face)**	Yes ☐<br>No ☐		
**General Practitioner (telephone)**	Yes ☐<br>No ☐		
**Out of hours contact (GP or deputy)**	Yes ☐<br>No ☐		
**Out of hours contact (Nurse)**	Yes ☐<br>No ☐		
**Practice Nurse (at the GP clinic)**	Yes ☐<br>No ☐		
**Other health professional involved with patients' mental health care (e.g. psychiatrist, community mental health worker)**	Yes ☐<br>No ☐		

If there is any other information or comments you have about the patient that you feel may be relevant to the study, please write them in the box below:

