# Supplementary material for: Predicting intentions towards long-term antidepressant use in the management of people with depression in primary care: A longitudinal survey study
Source: PLoS One. 2025 Mar 4;20(3):e0299676. doi: 10.1371/journal.pone.0299676 (PMC11878936; doi:10.1371/journal.pone.0299676)
Supplement: S1 Table — (PDF) [file pone.0299676.s004.pdf]

**S1 Table. Mean scores for beliefs about depression and antidepressant discontinuation**

| Variable | Items | N (%) | M | SD | 95% CI | | α |
|---|---|---|---|---|---|---|---|
| | | | | | Lower | Upper | |
| Intention | 3 | 272 (98.2%) | 2.44 | 1.78 | 2.23 | 2.65 | 0.91 |
| Attitude | 7 | 205 (74.0%) | 3.31 | 1.46 | 3.11 | 3.51 | 0.84 |
| Subjective norm | 4 | 252 (91.0%) | 2.35 | 1.15 | 2.21 | 2.49 | 0.70 |
| PBC | 3 | 238 (85.9%) | 3.78 | 1.20 | 3.63 | 3.94 | 0.53 |
| Necessity | 5 | 273 (98.6%) | 13.7 | 4.02 | 13.24 | 14.20 | 0.85 |
| Concerns | 5 | 273 (98.6%) | 8.09 | 4.38 | 7.57 | 8.61 | 0.82 |
| Cause | | | | | | | |
| Past events | 3 | 226 (81.6%) | 3.64 | 1.75 | 3.41 | 3.87 | 0.78 |
| Personal flaws | 4 | 208 (75.1%) | 3.59 | 1.28 | 3.42 | 3.77 | 0.58 |
| Physical causes | 3 | 212 (76.5%) | 2.88 | 1.40 | 2.69 | 3.07 | 0.49 |
| Bereavement | 1 | 244 (88.1%) | 3.26 | 2.14 | 2.99 | 3.53 | - |
| Timeline | | | | | | | |
| Chronic | 2 | 259 (93.5%) | 4.65 | 1.52 | 4.46 | 4.84 | 0.92 |
| Cyclical | 2 | 239 (86.3%) | 3.99 | 1.58 | 3.79 | 4.19 | 0.88 |
| Cure/control | | | | | | | |
| Talking therapy | 4 | 223 (80.5%) | 3.79 | 1.40 | 3.61 | 3.98 | 0.83 |
| Self-efficacy (thoughts) | 3 | 231 (83.4%) | 3.89 | 1.02 | 3.70 | 4.00 | 0.69 |
| Alternative therapy | 2 | 229 (82.7%) | 2.42 | 1.41 | 2.24 | 2.60 | 0.89 |
| Self-efficacy (behaviour) | 2 | 236 (85.2%) | 4.36 | 1.32 | 4.19 | 4.52 | 0.58 |
| Medication | 1 | 257 (92.8%) | 5.12 | 1.28 | 4.96 | 5.28 | - |
| Don’t know | 2 | 192 (69.3%) | 3.02 | 1.45 | 2.81 | 3.22 | 0.37 |
| Consequences | | | | | | | |
| Stigma | 3 | 238 (85.9%) | 3.91 | 1.37 | 3.73 | 4.08 | 0.68 |
| Avoidance | 3 | 243 (87.7%) | 3.70 | 1.64 | 3.49 | 3.91 | 0.86 |
| Spirituality/strength | 2 | 228 (82.3%) | 2.28 | 1.26 | 2.11 | 2.44 | 0.23 |

*Note*: α = Chronbach’s alpha for internal consistency between questionnaire survey items within each variable
