# Supplementary material for: Predicting intentions towards long-term antidepressant use in the management of people with depression in primary care: A longitudinal survey study
Source: PLoS One. 2025 Mar 4;20(3):e0299676. doi: 10.1371/journal.pone.0299676 (PMC11878936; doi:10.1371/journal.pone.0299676)
Supplement: S2 Table — (PDF) [file pone.0299676.s005.pdf]

S2 Table. Means, standard deviations, and intercorrelations for global beliefs on attitude

**Variables (N= 173)**	**1**	**2**	**3**	**4**	**5**	**6**	**M**	**SD**
1. Attitude	1.00	**-0.61**	**0.29**	**-0.43**	**-0.28**	**-0.46**	3.33	1.49
2. Necessity		1.00	**-0.15**	**0.32**	**-0.21**	**0.43**	13.69	3.88
3. Concerns			1.00	-0.46	-0.04	-0.12	8.32	4.30
4. Medication				1.00	0.10	**0.26**	5.09	1.25
5. Physical					1.00	**0.20**	2.91	1.37
6. Chronic						1.00	4.71	1.46

Note: Intercorrelations in bold are statistically significant.
