# Supplementary material for: Predicting intentions towards long-term antidepressant use in the management of people with depression in primary care: A longitudinal survey study
Source: PLoS One. 2025 Mar 4;20(3):e0299676. doi: 10.1371/journal.pone.0299676 (PMC11878936; doi:10.1371/journal.pone.0299676)
Supplement: S3 Table — (PDF) [file pone.0299676.s006.pdf]

S3 Table. Global beliefs associated with attitudes towards starting to come off antidepressants

**Attitude (N= 173)**	**B**	**95% for B**		**R^2**	**Adj. R^2**
		**(Lower)**	**(Upper)**		
Constant	**7.66**	6.48	8.84	0.50	**0.48**
Necessity	**-0.16**	-0.21	-0.12		
Concerns	0.04	-0.01	0.09		
Medication	-**0.21**	-0.40	-0.03		
Physical	**-0.15**	-0.28	-0.02		
Chronic	**-0.20**	-0.35	-0.05		

Note: Values in bold are statistically significant
