# Supplementary material for: Predicting intentions towards long-term antidepressant use in the management of people with depression in primary care: A longitudinal survey study
Source: PLoS One. 2025 Mar 4;20(3):e0299676. doi: 10.1371/journal.pone.0299676 (PMC11878936; doi:10.1371/journal.pone.0299676)
Supplement: S4 Table — (PDF) [file pone.0299676.s007.pdf]

S4 Table. Means, standard deviations and intercorrelations for beliefs and attitudes on intentions

**N= 161**	**1**	**2**	**3**	**4**	**5**	**6**	**7**	**8**	**9**	**10**	**11**	**12**	**13**	**14**	**M**	**(SD)**
1. Intention	1.00	**0.75**	**0.60**	**0.54**	**-0.47**	**0.42**	**-0.21**	**-0.46**	**-0.48**	**0.19**	-0.03	-0.05	-0.12	**-0.25**	2.62	1.87
2. Attitude		1.00	**0.55**	**0.59**	**-0.61**	**0.32**	**-0.26**	**-0.44**	**-0.45**	0.11	-0.05	**-0.15**	**-0.19**	**-0.28**	3.33	1.49
3. Subjective norm			1.00	**0.31**	**-0.38**	**0.35**	**-0.13**	**-0.35**	**-0.34**	-0.03	0.02	**-0.13**	0.00	**-0.21**	2.48	1.16
4. PBC				1.00	**-0.52**	0.11	**-0.16**	**-0.42**	**-0.31**	0.09	-0.03	**-0.22**	**-0.36**	**-0.33**	3.58	1.45
5. Necessity					1.00	**-0.15**	**0.21**	**0.43**	**0.32**	-0.10	0.03	**0.17**	**0.32**	**0.29**	13.65	3.93
6. Concern						1.00	-0.08	**-0.16**	**-0.46**	0.09	-0.13	-0.02	**0.29**	0.07	8.25	4.26
7. Physical							1.00	**0.16**	0.13	-0.01	0.01	-0.10	**0.17**	-0.01	2.90	1.35
8. Chronic								1.00	**0.29**	**-0.17**	-0.10	-0.10	**0.28**	**0.27**	4.73	1.46
9. Medication									1.00	-0.06	-0.02	-0.04	0.04	-0.01	5.07	1.28
10. With doctor										1.00	-0.07	0.29	0.08	0.05	1.53	0.50
11. Without doctor											1.00	**0.06**	-0.11	-0.06	1.60	0.49
12. Successfully stopped												1.00	0.10	**0.26**	1.69	0.46
13. Symptom severity													1.00	**0.17**	8.35	6.48
14. Current antidepressant duration (months)														1.00	125.08	101.71

Note: Values in bold are statistically significant
