# Supplementary material for: Predicting intentions towards long-term antidepressant use in the management of people with depression in primary care: A longitudinal survey study
Source: PLoS One. 2025 Mar 4;20(3):e0299676. doi: 10.1371/journal.pone.0299676 (PMC11878936; doi:10.1371/journal.pone.0299676)
Supplement: Attachment — Submitted filename: Response to Reviewers.pdf [file pone.0299676.s008.pdf]

Reviewer Comment	Response to Reviewers	Line
1. Please ensure that your manuscript meets PLOS ONE's style requirements, including those for file naming. The PLOS ONE style templates can be found at<br><br>https://journals.plos.org/plosone/s/file?id=wjVg/PLOSOne_formatting_sample_main_body.pdf and<br><br>https://journals.plos.org/plosone/s/file?id=ba62/PLOSOne_formatting_sample_title_authors_affiliations.pdf	We hope we have submitted the manuscript according to the style requirements for PLOS One.	
2. We note that you have indicated that there are restrictions to data sharing for this study. PLOS only allows data to be available upon request if there are legal or ethical restrictions on sharing data publicly. For more information on unacceptable data access restrictions, please see http://journals.plos.org/plosone/s/data-availability#loc-unacceptable-data-access-restrictions.<br><br>a) If there are ethical or legal restrictions on sharing a de-identified data set, please explain them in detail (e.g., data contain potentially identifying or sensitive patient information, data are owned by a third-party organization, etc.) and who has imposed them (e.g., a Research Ethics Committee or Institutional Review Board, etc.). Please also provide contact information for a data access committee, ethics	Thank you for providing a clear outline of the data sharing policy with us.<br><br>Unfortunately, there are ethical restrictions in place for sharing the data publicly, despite the data being de-identified. The data were collected as part of the corresponding author's PhD candidature (https://eprints.soton.ac.uk/475885/). As part of the Ethics Review Process, the IRAS Submission Form (Section A51, Page 20) asked how the results of the study were to be reported and disseminated. The statement 'Access to raw data and right to publish freely by all investigators in study	

committee, or other institutional body to which data requests may be sent.<br><br>b) If there are no restrictions, please upload the minimal anonymized data set necessary to replicate your study findings to a stable, public repository and provide us with the relevant URLs, DOIs, or accession numbers. For a list of recommended repositories, please see<br><br>https://journals.plos.org/plosone/s/recommended-repositories. You also have the option of uploading the data as Supporting Information files, but we would recommend depositing data directly to a data repository if possible.<br><br>We will update your Data Availability statement on your behalf to reflect the information you provide.	or by Independent Steering Committee on behalf of all investigators' was NOT selected.<br><br>Furthermore, participants did not provide any consent for their data to be shared anonymously for other ethically approved research in the future.<br><br>The corresponding author has submitted the IRAS form and University of Southampton's Thesis Licence form are included as part of our resubmission to show the restrictions from both the NHS Ethics Committee and University of Southampton (as the host institution for RDH's PhD Candidature).	
Reviewer #1: Statistical methods - Why was hierarchical regression modelling used? Why was multiple linear regression with robust SE used for some analyses and hierarchical models for others? This needs an explanation/ better write up of statistical methods.	The literature suggests that underlying salient beliefs may determine attitudes towards a behaviour (see Conner & Armitage, 1998). We wanted to determine whether salient beliefs about depression and antidepressant use could determine attitudes towards starting to come off antidepressants, so we conducted a multiple linear regression analysis to investigate this. This was separate to the hierarchical regression analysis which was conducted to	

	determine how well the original constructs of the TPB model predicted intentions to start to come off antidepressants, then investigated whether adding other theoretical constructs, in order of priority, would strengthen the model.<br><br>For clarity, we have added a statement in the section ‘Outcomes and variables’ to justify why we conducted these two analyses.<br><br>We have also given further explanation in the ‘Statistical methods’ section<br>to outline the steps taken in the linear regression and hierarchical regression analyses.	146-148<br><br>232-242
Lines 237-238 and 247 and elsewhere - present IQR as lower quartile, upper quartile.	Thank you for highlighting this reporting error. We have now changed the data to show the lower and upper quartiles at each relevant place in the manuscript.	283, 292

Table 3 - If you are going to present mean, median, (SD) present in this order mean (SD), median (LQ, UQ)	Thank you for highlighting this reporting error. We have now made the amendments to Table 3 to present the data as mean (SD), median (LQ, UQ).	Table 3
Line 269 - I would not focus on the p-value, especially as the correlation coefficient is so small. Please remove the p-value.	We agree with the reviewer that the p-value is not terribly informative in the context of interpreting the correlation coefficients. However, the journal reporting guidelines seem to prefer that these are included. We are happy to remove these if this is acceptable to the editor.	278
Lines 286-287 - along with mean and SD does not seem to fit in this sentence.	For clarity, 'along with the mean and standard deviations' has been removed from the end of the sentence, and replaced by a new sentence stating "The means, standard deviations, and intercorrelations for salient beliefs on attitude are presented in S2_Table"	330-332
Lines 292-293 - reword to take slope coefficient out.	We have removed 'The slope coefficients show that' from the beginning of the sentence.	
Line 297 - coefficient and 95%Ci would be more useful.	We have provided the coefficient and 95% confidence interval to show the contribution of necessity of antidepressants towards attitudes around discontinuation.	340
Is line 301 a repeat of line 290? If so, remove one.	Thank you for this comment. We have now amended this finding to report:	332-334

	"The multiple correlation coefficient (*R*= 0.71) showed a moderate to strong linear relationship between salient beliefs and attitudes towards stopping antidepressants *F*(5, 167)= 33.03, *p*<0.001, adj. R^2= 0.48."	
Line 326 and elsewhere - If you are going to include p-values (would prefer it if they are not included), please give exact p-values for p-values greater than or equal to 0.001.	As with the previous response to reporting *p* values, we agree that these are not necessarily informative to the reader compared to the reporting of Confidence intervals. We have adjusted any *p* value greater than 0.001 to report the exact figure, and have left them in the text for now. However, if the editor is in agreement with the reviewer, we are happy to remove the *p* values from the manuscript.	
Table 5 needs some explaining. I assume 95%CI is for step 5.<br>Table 5 - I am not sure what the column on the far right is.<br>Table 5 - I would remove stars to signify statistical significance and add 95%CI for all models so that the reader can make their own judgement on the importance of each variable.<br>Table 5 - Also please make it clearer what the four rows at the bottom of the table are showing.	Thank you for your feedback regarding the presentation of the data in Table 5. We have reconfigured the table to present the data more clearly. As with previous comments, we have left *p* values in the table and emboldened the significant values. However, if the editor is happy to remove the *p* values (as the 95% CIs for *B* are included for each step), then we agree that the *p* value columns can be removed to allow the reader to make their own inferences.	Table 5

Table 5 Why include robust SE beta? (again, changes to the statistical methods will help with this one).	We have restructured Table 5 to present the data from the hierarchical regression more clearly. For clarity, we have also added the following statement to the Statistical methods section:<br><br>'Tests to ensure the data met the assumptions for multivariate analysis found heteroscedasticity of residuals; therefore parameter estimates with robust standard errors were calculated to account for this heteroscedasticity'.	Table 5<br><br>232 -234
Table 5 - check the constant for step 2.	We have checked the constant for step 2 and it is correctly reported as -.55.	Table 5
Table 5 and elsewhere - you cannot predict an outcome from data collected at the same time. Please change the terminology to something like factors associated with. Prediction can be used for outcomes that come from the notes as they represent a later time point than the survey.	Thank you for this comment. We have changed the term 'predict' when discussing intentions as the outcome to use 'factors associated with' or 'to explain' where appropriate.	Highlighted throughout manuscript
Table 6 and explanation below - It looks like you have carried out a non-parametric test, but have presented mean and (SD). If you carry out a non-parametric test, then please give median (LQ, UQ) for descriptive statistics.	We agree that this table is confusing for the reader. We have removed it and reported on the median scores between the two groups as part of the non-parametric test.	374 - 380
Page 24, 2nd line - change expB to odds ratio.	We have changed expB to OR	390

Table S1 - please explain what the column at the far right of the table is showing. It is not clear.	The last column presents Chronbach's alpha for internal consistency between the questionnaire survey items for each variable. We have added a note for clarity at the bottom of Table_S1.	Table S_1
Table S2 - please remove stars to represent levels of statistical significance.	In line with the other comments from the reviewer regarding the reporting of levels of statistical significance, we have removed the stars, but have emboldened any intercorrelations that are significant, for consistency with our other amendments. We welcome the final decision regarding the presentation of statistical significance from the Editor.	Table S_2
Table S3 - what is the difference between the three beta coefficients in this table? If all are needed, please ensure they are accompanied by 95%CI.	We have amended the table to only show one Beta Coefficient.	Table S_3
Table S4 - the last row seems to have been omitted.	Thank you for highlighting this oversight. We have now added Row 14 (Current antidepressant duration (months))	Table S_4
Reviewer #2: A very important piece of work contributing to the literature about understanding people's long-term antidepressant medication use and factors underlying their intention to considering stopping them. The framework used to explore the determinants of intention to stop medication was appropriate and it was followed by examining actual behaviour. This could be elucidating in terms of	We would like to thank the reviewer for their kind comments.	

understanding any gaps between behavioural intention and behaviour, factors that might get in the way and how to overcome them in clinical practice.		
Abstract<br>Well-presented and clearly indicates the rationale and main findings of the study.<br>The abstract states that normative beliefs, and not subjective norms, were one of the stronger predictors of intention. Please clarify whether it was normative beliefs or subjective norms.	Thank you. We have changed the term to subjective norms and we define the TPB variables in more detail in the methods section.	37<br>170-172
Although all TPB constructs and concerns about antidepressant use maintained their ability to predict intentions towards starting to come off antidepressants in the analysis, only "normative beliefs" and attitudes were mentioned in the abstract. Please mention all significant predictors.	We have amended the abstract results section to include the following: "All Theory of Planned Behaviour constructs and concerns were associated with intentions, with more favourable attitudes towards stopping and subjective norms the strongest predictors of intentions to discontinue antidepressant use."	42-45
Introduction Concise and well-written. Authors clearly explain the rationale of the study.	Thank you for this kind comment about the introduction.	
46 – Please clarify whether what was measured was normative beliefs and not actually subjective norms	We have changed the term to subjective norms.	Throughout manuscript
Methods<br>99-100- Please justify the decision about the inclusion criterion around duration of current antidepressant medication (over two years)	We have included a sentence that the decision to have a treatment duration of 2 years or longer is in line with the NICE guidance that suggests that individuals who have had	112-116

	recurrent episodes of depression could decide whether their GP whether to stop or continue antidepressant treatment after this time.	
121 – Please also name in the text the TPB variables that are hypothesised to predict the outcomes of interest.	We have added 'In addition to the TPB constructs of attitude, subjective norms, and perceived behavioural control,…' before describing the additional constructs that were included in the model.	142-143
123 – Error in the citation. Please correct this.	The formatting error has been corrected. Fig 1 is now appropriately referenced to the Fig 1 Caption.	Fig 1
139 - Please refer to the number of items used for each direct TBP variable (attitudes, subjective norms, perceived behavioural control, intention).	The number of items included in each variable is included in Supplementary Table 1. We have additionally added a column to Table 1 in the manuscript to include the number of items for each variable and refer the reader to this in the manuscript.	Table S_1<br>Table 1<br>215
144 - I would suggest rephrasing from good to acceptable as Cronbach's α of 0.60 is not very high and many sources would say that only values over 0.70 are acceptable (e.g. Cortina, J. M. (1993). What is coefficient alpha? An examination of theory and applications. Journal of applied psychology, 78(1), 98).	We agree with the reviewer's comment and have changed the phrasing from 'good' to 'acceptable'	175
145-147 - Please provide further description of the "composite variables for direct measures". Please clarify whether the items used tapped into the belief (indirect) measures of TPB or whether they	Thank you for this comment regarding the inconsistency in our reporting of subjective norms and normative beliefs. We have carefully considered the reviewers' comments around	169-172

measured the direct measures directly (subjective norms, attitudes, perceived behavioural control) as this is not clear in the Methods. In TPB there are direct (subjective norms, attitudes, perceived behavioural control) and indirect measures (behavioural beliefs, normative beliefs, control beliefs) that are determinants of behavioural intention. They are not synonymous. Please provide a brief definition for attitudes, subjective norms and perceived behavioural control either as part of the Method section or in the Introduction when mentioning TPB as the framework that was used.	the use of subjective norms and normative beliefs throughout the manuscript and acknowledge that it lacked clarity for the reader. We have revisited the literature and conclude that the items used closely align with the definition of 'injunctive normative beliefs' to measure subjective norms (ref: Ajzen's 2020 Theoretical Article (The theory of planned behavior: Frequently asked questions (wiley.com)). We have explained how each of the components from the TPB framework were considered and defined within the context of our study.	164-173
Please provide internal consistency estimates for each of the subscales (for each TPB determinant and intention). In case any of the items were dropped during item reliability testing, please report this.	The internal consistencies for the TPB determinants and intention are presented in Table_S1.	Table S1
148-149 - Please describe the constructs captured by PATD questionnaire as well as the number of items, scoring, min-max score. Please provide the internal consistency/reliability estimate for the present sample.	The version of the PATD that was used in the creation of the questionnaire is exploratory by design, therefore there is no scoring system s11096-012-9704-5.pdf (springer.com). We have included a sentence to say that the data from the items are presented as frequency scores. Items relating to past history of stopping antidpepressants are dichotomous	179-182

	and we have explained this in more detail in the relevant section.	
153 – Perhaps italicise “necessity” and “concerns” as you have done that a few lines further down for the other subscale of the measures to be consistent in the way of presenting information. Please provide the internal consistency/reliability estimate for the present sample for this measure and its subscales.	We have italicised ‘necessity’ and ‘concerns for consistency. The internal consistency estimates for this sample are presented in Table_S1.	186
150 - In TPB terms “salient beliefs” or readily accessible beliefs refer to the specific beliefs about the specific behaviour that is being addressed, in this case starting to come off antidepressant medication in the next 6 months. These are categorised in normative, behavioural and control beliefs about the specific behaviour. In this study, it seems that salient beliefs refer to more global beliefs about antidepressant medication or depression, and not specific to the behaviour of interest within the timeframe of interest. It might be worth rephrasing this section and wherever else the constructs captured by this questionnaire are mentioned, as “salient beliefs” commonly refers to something different in studies using a TPB framework.	We acknowledge this comment from the reviewer and their view on the use of the term of ‘salient beliefs’. We agree that it is sensible to change the term ‘Salient Beliefs’. We propose using the term ‘global beliefs’ and have made the necessary changes. We hope this term is more acceptable to the reviewer.	Throughout manuscript
160 – When referring to past behaviour, please clarify what these behaviours were in the text.	We have clarified what these behaviours were in the text – past behaviour was measured through three items – whether participants had stopped antidepressants in the past with/without their doctor’s knowledge, and whether	194-198

	they had successfully stopped taking antidepressants in the past.	
161-162- Please indicate the unit of measurement for the current treatment duration (e.g. months, years, etc)	We have edited this sentence to show that participants were asked to self-report their antidepressant duration in years and months	199
Table 1 Please correct typo – Subjective norms	The typo has been corrected	Table 1
169 – Please specify at what point the medical professionals at the GP surgeries reviewed participants records (e.g., at least 6 months after participation, looking through notes for the 6-month period following participation to the study)	We have specified at the start of this paragraph that medical notes reviews were carried out for the six-month period following participation to the study.	207-208
191-193 - Please provide rationale for conducting the Pearson' s correlation between necessity and concern around antidepressant medication.	We have revisited the literature and agree that there is little rationale for conducting a Pearson's correlation between necessity and concern around antidepressant medication. We have therefore removed this calculation.	
191- Please correct typo – Pearson's	The sentence has now been removed.	
195 – Please use lower care letter- "Salient"	We have now changed 'salient' to 'global' (see comments above)	
204 - "Transformation of the variables" is mentioned with no previous indication to what this refers to and what analysis was conducted regarding this. Please provide clarification on the transformation of any variables in the study and the reason for doing so.	While running the tests for assumptions for the logistic regression, we found that 9 of the 12 participants had studentized residuals ±2.5, meaning that the data did not meet the assumption that there should be no significant outliers, therefore did not fit the model well. We therefore	251-252

	attempted to transform the variables of intention and PBC scores for participants to correct for their non-normal distributions, but this did not correct the data. This meant it was not possible to conduct a logistic regression as the parametric assumptions were not met, despite attempts to transform the variables. We have put a sentence to say 'Therefore parametric assumptions to conduct a logistic regression were not met' and removed 'transformation of variables' for clarity, while keeping in the sentence explaining outliers based on inspection of the data.	
Results<br>245 – It is not clear what the authors meant by "successfully stopping" medication. Please clarify if that was defined using specific criteria in the questionnaire.	In the questionnaire, participants were given a definition of 'Successfully stopped' as 'symptom free episode(s) while off antidepressant treatment'. This has been clarified in the methods section that defines Past behaviour.	197-198
272-282 – It is unclear whether these findings relate to yes/no questions. For example, is it that 24.2% responded that they are certain about stopping taking their antidepressants and they authors have interpreted this as the majority being uncertain? Please clarify what these percentages refer to as it is not clear.	We have described the items used from the PATD in more detail on to show that items were 5 point Likert Scales, with no scoring system. Therefore frequency data are shown in the results section. We have amended the reporting of the frequency data to show how many participants gave responses (e.g. 'strongly agreed' or 'agreed') for clarity	179-182<br><br>315-322

Discussion<br>Line numbering stopped at page 23.	Apologies for the line-numbering error – this was due to a section break inserted in the manuscript. Line numbering has been included after this section break.	
First paragraph: The way this is presented is that TPB was used and applied to the study to help identify factors that might determine participants' intention (and actual behaviour). The last sentence is somewhat contradictory, as it is stating that the TPB was applied after measuring participants' beliefs and attitudes to see if it could explain them. Please rephrase for clarity.	We have removed the final sentence and amended the first sentence to say that we used an extended model of the TPB.	393-395
Second paragraph: Here, normative beliefs are mentioned as a significant predictor of intention. However, in the model shown in figure 2 normative beliefs are not included. Please clarify. It is important to note that in TPB terminology "normative beliefs" (refers to indirect measure of TPB and different items are used to measure this) is a different construct to "subjective norms" (direct measure of TPB).	Thank you for highlighting this. As with our previous comment, we have changed the terminology to 'subjective norm' for consistency	
Third paragraph<br>1st line: please correct grammar error – "felt taking medication was necessary".	We have changed the grammatical error.	402
Here, it stated that "necessity" was the most important predictor when considering stopping antidepressant medication. From the analysis and results, it seems that necessity was linked with attitudes	We have changed this sentence for better clarity to say 'Necessity beliefs about antidepressants appeared to be the	404-405

towards coming off the medication however, it seems that they did not contribute significantly towards the intention of doing it. Please consider how this might be the case and be more precise in the interpretation of this result as “considering” stopping the medication seems to relate more to the intention rather than the favourable/ unfavourable attitudes towards stopping the medication.	most important factor associated with attitudes towards starting to come off antidepressants’	
Fourth paragraph (page 25 of the submitted manuscript): It is mentioned that subjective norms significantly predicted intention. However, the next sentence refers to results in other items and specifically, items on page 8 and item 33, which was not a subjective norm item. Although this could help further explain the significant effect of subjective norms being a significant predictor of intention, it is not the same thing. Please discuss this finding in relation to existing relevant literature around subjective norms and their importance on predicting intention of coming off antidepressants. It is also important to discuss the difference between subjective norms that refer to a specific behaviour (coming off antidepressants in the next 6 months) in comparison to general attitudes about being comfortable to stop medication if doctors would support participants to do so at some point in the future if it was discussed. Additionally, subjective norms do not only refer to their doctor’s views about stopping medication but also to “most people important” to the participant, “people who	We have added a sentence to highlight some of the key evidence regarding views of significant others on patients’ use of antidepressants, as well as the potential role of significant others in predicting intentions towards stopping antidepressant use.	416-420

care about " the participant, and the social pressure more broadly. Considering the intention to stop medication was low on average, it might be worth considering how other people's views might impact participants' intention.		
Implications for primary care: The first paragraph summarises relevant literature that builds the rationale for conducting this study, which would be more appropriate for an introduction section, rather than linking clearly specific findings from this research to clinical practice. Please refer to the implications with clear reference to the specific findings of this study (e.g., importance of necessity beliefs that are linked to attitudes, which in turn were significant predictors of intention).	We have moved the first paragraph to the introduction section as we agree this strengthens the rationale for the study.<br><br>We have referred to the implications of having a better understanding of the long-term risk/benefits towards antidepressant use and how these beliefs could link to attitude and subsequent discontinuation.	66-78<br><br>518-522
Pages 6 and 7 of the APPLAUD questionnaire seem to refer to "indirect" measures of TPB (belief items) that were not mentioned in the Methods section of the manuscript, and it is unclear if they were used in the analysis and how. Only the direct measures of TPB have been included in the analysis model as presented by the authors. Please clarify.	The items on Page 7 of the APPLAUD questionnaire are items adapted from the PATD questionnaire. As the PATD questionnaire is exploratory by design, the items were not included in the TPB model as items were not scored. They are discussed in the 'Beliefs about depression and antidepressant discontinuation section' of the Results section.	315-327
