# Supplementary material for: Predicting intentions towards long-term antidepressant use in the management of people with depression in primary care: A longitudinal survey study
Source: PLoS One. 2025 Mar 4;20(3):e0299676. doi: 10.1371/journal.pone.0299676 (PMC11878936; doi:10.1371/journal.pone.0299676)
Supplement: Attachment — Submitted filename: Response_to_Reviewers_auresp_2.pdf [file pone.0299676.s009.pdf]

Reviewer Comment	Response to Reviewers
Line 235 - change multivariate to multivariable. They have different meanings and you mean multivariable. https://bjo.bmj.com/content/101/10/1303	Thank you for highlighting this error and for taking the time to include a link to the BJO paper. We have changed the term multivariate to multivariable (Line 235).
Line 240 - I am not sure that what you are describing is hierarchical regression. I think you are just adding variables sequentially to a linear regression model. If you are using hierarchical modelling, can you state what the random effect is in the methods.	Thank you for this comment. For our study, the term 'hierarchical regression' was used to explain that variables were added in a sequential fashion, but prioritised according to the theoretical importance of each variable. We appreciate that this is confusing and have referred back to Tabachnick & Fidell's work that states that 'sequential regression' is sometimes referred to 'hierarchical regression'. To avoid confusion, we have changed 'hierarchical' to 'sequential' (Line 240).
Line 319 - Should 134 be 234 to make the percentage make sense? I assume the n~277.	Thank you for highlighting this typo. We have double-checked the data and it is 234 participants and have made the correction (Line 319).
Line 323 - is 24.2 a percentage?	Thank you for spotting this. Yes, this is a percentage – we have added the missing % (Line 323).
Line 343 - 0.16 should be -0.16.	Thank you again for spotting this. We have added a negative symbol. (Line 343)
Line 343 - The p-value is not 0.00. Increase the number of decimal places or use p<0.01.	As the p-value would need to be reported to 10 decimal places (p= 0.000000000179…) we hope that it is acceptable to report the p value as <0.01 (Line 343).
Thank you for the opportunity to review this important paper. This paper covers the important topic of long-term antidepressant use in primary care	We would like to thank the reviewer for their very kind comments about our paper.

patients. The study is well-motivated with the relevant scientific literature, and well-conducted with an underlying theory and adequately adapted questionnaires. The study is very relevant, certainly for primary care, the site with the most antidepressant users. The study quantitatively extends knowledge from previous qualitative studies.	
1. Implications for primary care: In my opinion the practical value of the paper improves with making a distinction between starting an antidepressant and how to manage follow-up prescriptions. The authors have shown that stronger beliefs in the necessity of antidepressants, stronger beliefs that depression can be cured with antidepressants, that depression has a physical cause and is chronic, are all related with more negative attitudes towards discontinuation. These beliefs are certainly also induced by prescribing GPs. GPs should take care of avoiding to mention these issues but instead stress the social causes of depression, the fact that medication can be helpful but is to be considered only part of the treatment alongside lifestyle adaptations and conversations about the context of depression. Moreover, GPs should always stress the limited duration of antidepressant use. For the follow-up prescriptions, GPs should be clear about scheduled face-to-face consultations and so on (as the authors already describe in the paper.)	Thank you for your comments. We have added a sentence (Lines 516 – 519) to emphasise the need to discuss psychosocial causes of depression along with lifestyle adaptations at the point of prescribing antidepressants. We have also added ‘the limitation duration of antidepressant use’ (Line 520) to highlight your second point. We hope this strengthens the implications section of the discussion.<br>These implications are explored (and explained) further by the qualitative findings from this mixed-methods study, and RDH is preparing the manuscript for publication in due course.

2. May be this is a personal preference, but I would like a separate description of the main results of the study and the comparison with the literature. Now, these paragraphs seem to overlap.	Thank you for this comment – we have considered structuring the discussion in the way you suggested, but feel that this could extend the length of the manuscript, which is already relatively long. We will therefore leave the discussion section in its current structure; however we welcome any decision from the editors regarding this.
1. The study title indicates that this is a longitudinal study while in the Methods (page 6 line 105 the authors use the term cross-sectional.	We agree that this is confusing for the reader – we have changed Line 105 to define the study as 'longitudinal'.
2. I'm a bit confused about the numbers: (a) on page 14 line 268 there are 189 participants of whom medical data were received, while on page 19 line 298 prescribing outcomes were obtained for 175 participants and on page 20 line 332 multiple linear regression was performed for 173 participants. This is probably due to missing responses. Maybe a short statement about this is sufficient.	Thank you for this observation and we understand that this may be confusing for the reader. We do mention in the 'Statistical methods' section that data analysis was conducted using complete cases. We have added "(i.e. complete cases)" to Line 333 to clarify why n=173. We also acknowledge a key limitation of the study was the level of missing data, despite testing the face validity of the questionnaire prior to its implementation in the current study (Lines 477-484).<br><br>We contacted all 20 practices to request notes review data for all participants who were entered into the trial (n= 277) – we received notes review data for 189 participants, with the reasons for the outstanding 88 participants mentioned in Lines 268-272. We have amended the statement in Line 298-299 to say that prescribing outcomes were recorded at 6 months for 175 participants (92.6% of participants with notes review data) for clarity.

(b) in Figure 2 the numbers are not correct: in the box with excluded participants, the numbers for the different reasons for exclusion do not add up to 119	Thank you for highlighting this – when converting the table to a TIF file, the last two exclusion criteria (participant no longer at address and blank questionnaire returned) were cut from the image. The TIF image has been corrected.
